# Synergistic integration of Multi-View Brain Networks and advanced machine learning techniques for auditory disorders diagnostics

**DOI:** 10.1186/s40708-023-00214-7

**Published:** 2024-01-14

**Authors:** Muhammad Atta Othman Ahmed, Yasser Abdel Satar, Eed M. Darwish, Elnomery A. Zanaty

**Affiliations:** 1 Department of Computer Science, Faculty of Computers and Information, Luxor University, 85951 Luxor, Egypt; 2 Mathematics Department, Faculty of Science, Sohag University, 82511 Sohag, Egypt; 3 Physics Department, College of Science, Taibah University, Medina, 41411 Saudi Arabia; 4 Physics Department, Faculty of Science, Sohag University, 82524 Sohag, Egypt; 5 Department of Computer Science, Faculty of Computers and Artificial Intelligence, Sohag University, 82511 Sohag, Egypt

**Keywords:** Neurological disorders, Auditory impairments, Deafness, Tinnitus, Multi-View Brain Networks, EEG-based diagnosis, Ensemble learning, Feature reduction, Diagnostic modeling

## Abstract

In the field of audiology, achieving accurate discrimination of auditory impairments remains a formidable challenge. Conditions such as deafness and tinnitus exert a substantial impact on patients’ overall quality of life, emphasizing the urgent need for precise and efficient classification methods. This study introduces an innovative approach, utilizing Multi-View Brain Network data acquired from three distinct cohorts: 51 deaf patients, 54 with tinnitus, and 42 normal controls. Electroencephalogram (EEG) recording data were meticulously collected, focusing on 70 electrodes attached to an end-to-end key with 10 regions of interest (ROI). This data is synergistically integrated with machine learning algorithms. To tackle the inherently high-dimensional nature of brain connectivity data, principal component analysis (PCA) is employed for feature reduction, enhancing interpretability. The proposed approach undergoes evaluation using ensemble learning techniques, including Random Forest, Extra Trees, Gradient Boosting, and CatBoost. The performance of the proposed models is scrutinized across a comprehensive set of metrics, encompassing cross-validation accuracy (CVA), precision, recall, F1-score, Kappa, and Matthews correlation coefficient (MCC). The proposed models demonstrate statistical significance and effectively diagnose auditory disorders, contributing to early detection and personalized treatment, thereby enhancing patient outcomes and quality of life. Notably, they exhibit reliability and robustness, characterized by high Kappa and MCC values. This research represents a significant advancement in the intersection of audiology, neuroimaging, and machine learning, with transformative implications for clinical practice and care.

## Introduction

Audiological disorders, including deafness and tinnitus, represent significant challenges to individuals and healthcare systems worldwide [1]. These conditions can lead to profound and often debilitating consequences, affecting auditory perception and overall quality of life. Deafness, complete loss of hearing, and tinnitus, the perception of phantom sounds are major health concerns [2]. Identifying auditory disorders early is key to personalized treatment and better outcomes [3]. Traditional methods for diagnosing hearing disorders are limited due to subjective assessments and variability in interpretation. As a result, there is a growing interest in leveraging advanced neuroimaging techniques and machine learning approaches to provide more objective and accurate means of diagnosis. Integrating Multi-View Brain Network data with state-of-the-art machine learning algorithms shows promising results [4]. This organ is the product of successful evolution, allowing us to perceive, understand, and interact with the world in ways that surpass all other species [5]. The brain’s intricate network of neurons, synapses, and signaling pathways directs human cognition and behavior [6]. The brain networks are like a massive communication network, with interconnected areas constantly transmitting signals [7]. Neural networks sustain our consciousness, emotions, and memories, creating unique human experiences. These networks intricately weave together to create a tapestry that sets us apart as unique individuals [8]. Advanced technologies are crucial for neuroscience to comprehend the intricacies of neural networks, such as functional magnetic resonance imaging (fMRI) and EEG, to peek into the real-time dynamics of brain activity [9]. Brain network analysis tools reveal how our cognitive and emotional faculties work. The study of connections between neurons and humans provides insight into consciousness, self-determination, and human nature [10].

Deafness is a sensory impairment that exerts a considerable impact on the organization of human brain networks, leading to neuroplasticity [11]. Changes in sensory input or experiences can trigger the brain’s reorganization ability. This is especially true for individuals who experience hearing loss, such as those who are deaf. As a result, they often possess superior visual processing skills, including improved visual acuity, motion detection, and spatial abilities [12]. Changes in connectivity among different brain regions, including the left hemisphere responsible for sign language processing, occur in patients with hearing loss due to the reorganization of brain networks [13]. Acknowledging sign language as a legitimate language significantly influences the brain regions that manage language processing. The cognitive system responsible for short-term memory and information manipulation is often enhanced in deaf individuals [14]. Hearing loss can have a significant impact on social and emotional processing. It is widely acknowledged that people with normal hearing process emotional signals differently than those with hearing impairment [15]. Understanding neural adaptations and developing targeted interventions and assistive technologies are essential to improve the quality of life for individuals with hearing impairments [16]. Tinnitus is a common medical condition that causes individuals to experience sounds in their ears even when there is no external auditory stimulus. This condition affects millions worldwide and should not be taken lightly [17]. Tinnitus has many factors that interact in complex ways, leading to its cause. Although the cause may not always be clear, it is believed that alterations in brain networks could play a role in its development [18]. Any changes within the auditory system will undoubtedly affect its ability to hear and process sound effectively [19].

Machine learning in audiology is indispensable for the Automated Diagnosis of auditory disorders through Multi-View Brain Networks. With their remarkable ability to process vast amounts of data and identify intricate patterns, Machine Learning algorithms are highly effective in accurately and efficiently classifying auditory disorders [20].

The primary objective of this study is to create a system that can improve the diagnosis of auditory disorders such as deafness and tinnitus. This is achieved by analyzing the complex relationships between different brain regions using a clustering coefficient based on triangle motifs. The feature reduction technique PCA is utilized to manage the high-dimensional nature of brain connectivity data. The study employs ensemble learning techniques to produce accurate and reliable predictive models. The main contributions of this work can be summarized as follows:

Use machine learning for early and accurate diagnosis of deafness and tinnitus to enhance patient outcomes and quality of life. Apply Multi-View data and combine different views in EEG data and 10 ROI to achieve better diagnosis results. Build a Robust model by leveraging the strengths of ensemble learning algorithms. Assess the effectiveness of classification findings by evaluating proposed models using a variety of metrics. Compare our findings results with existing state-of-the-art approaches. The remaining sections of this paper are arranged in the following: Sect. 2 provides a comprehensive overview of pertinent research concerning identifying auditory disorders through Multi-View Brain Networks. Section 3 covers the proposed research methodology, which includes the creation of multi-view brain networks, exploratory data analysis, and data visualization. In section 5, the details of our experimental hypothesis modeling and setup are outlined. The experimental findings and results are detailed in Sect. 6. Section 7 gives the work conclusion and recommendations for future work that can be implemented to achieve positive outcomes.

## Related work

In recent years, the intersection of audiology, neuroimaging, and machine learning has prompted various investigations to advance the understanding and diagnosis of auditory disorders. A survey of relevant literature reveals several pertinent studies that contribute to developing similar methodologies.

Chen et al. [21] explored the application of fMRI data in characterizing functional brain network alterations associated with tinnitus. Their study underscored the utility of resting-state fMRI data in identifying distinctive connectivity patterns within the auditory networks of tinnitus patients.

Smith and Jones [22] conducted an extensive review of neuroimaging studies focused on deafness-related plasticity in the auditory cortex. Their synthesis highlighted the remarkable capacity of the brain to reorganize neural pathways in response to auditory deficits, a phenomenon contributing to the establishment of novel diagnostic frameworks.

In machine learning, Li et al. [23] explored the efficacy of Support Vector Machines in classifying individuals with tinnitus based on their neuroimaging profiles. Their results demonstrated promising classification accuracy, motivating further exploration of diverse machine learning techniques as we undertake in this study.

Moreover, Johnson et al. [24] intersects with our methodology by utilizing Multi-View Brain Network data to differentiate neurological disorders. While not confined to auditory disorders, their successful integration of multi-view data offers a model for a multi-modal approach.

EEG data were used in [25] to build a brain networks model and detect functional connectivity patterns for individuals with auditory disorders. By analyzing functional connectivity in brain networks, another study [26] sought to differentiate between prelingually deaf infants with and without cochlear implants. Using a novel method for dividing regions of interest (ROIs), the study obtained significant enhancements in classification accuracy.

In addition, machine learning has been used to predict normal and pathological phenotypes from large-scale human brain networks by comparing various brain network kernels for classification purposes [27]. Moreover, functional near-infrared spectroscopy (fNIRS) and machine learning have been used to differentiate individuals with and without tinnitus, with significant differences between tinnitus patients and controls in resting-state measures of connectivity and evoked responses [28]. While fMRI studies have examined brain activation in tinnitus patients, cognitive control, and default mode networks may be involved in non-auditory aspects of the disorder [29].

The structure of the human cerebral cortex can be estimated using intrinsic functional connectivity. Using resting-state functional connectivity magnetic resonance imaging (MRI), the configuration of networks in the human cerebrum was investigated [30]. Local networks confined to the sensory and motor cortices and distributed networks of association regions were discovered. Functional connectivity within the sensory and motor cortices followed topographic representations across adjacent areas, whereas connectivity patterns in the association cortex frequently exhibited abrupt network boundary transitions [31]. According to another study, three interdependent architectural gradients underline the organization of intrinsic functional connectivity in the human cerebral cortex. These gradients correlated with external versus internal information sources, content representation versus attentional modulation, and central versus peripheral brain regions [32]. In addition, intrinsic functional connectivity MRI was used to compare rodent and human cortico-hippocampal connectivity. The results demonstrated preferential connectivity of sensory cortical networks in rats, as opposed to association cortical networks in humans [33].

Using machine learning techniques, human brain networks can be analyzed [34]. These techniques employ various algorithms, including K-Nearest Neighbor, Support Vector Machine, and Artificial Neural Network, to analyze brain images and identify connectivity patterns [35]. Machine learning methods can also be used to develop fMRI network inference methods, such as BrainNET, which quantify the contributions of various brain regions [36]. Deep learning techniques, such as Graph AuTo-Encoding (GATE), have been devised to characterize the population distribution of brain graphs and infer their relationships with human characteristics [37]. In addition, deep learning methods have been applied to classify brain networks for detecting Alzheimer’s disease (AD) [38]. Several statistical and machine learning link selection methods have been evaluated for brain functional networks, resulting in better utilization of network representations. Multimodal neuroimaging can present valuable information in the diagnosis of dementia. However, the small size of complete multi-modal data limits the ability of representation learning. In Ref. [39], the authors proposed a novel framework for the AD diagnosis called Multimodal-Representation-Learning and Adversarial Hypergraph-Fusion. This framework combines distribution-based GraphGAN and CNN-based GraphAE to extract features in the representation space. An adversarial strategy is utilized in modal fusion to improve the accuracy of AD detection. Results obtained on the ADNI dataset show that prior information can help enhance discrimination of representation learning. Also, adding more modalities can improve the detection performance.

In Ref. [40], the authors proposed a novel Consistent Perception Generative Adversarial Network (CPGAN) for semi-supervised stroke lesion segmentation. The proposed CPGAN can reduce the reliance on fully labeled samples. Specifically, a Similarity Connection Module (SCM) was designed to capture the information of multi-scale features. The proposed SCM can selectively aggregate the features at each position by a weighted sum. An assistant network was constructed using a consistent perception strategy to improve meaningful feature representation learning to enhance brain stroke lesion prediction accuracy for unlabeled data. They employed the assistant network and the discriminator to decide whether the segmentation results were real or fake. The CPGAN was evaluated on the Anatomical Tracings of Lesions After Stroke (ATLAS). The experimental results demonstrated that the proposed network achieves superior segmentation performance.

## Proposed research methodology

### Data insights

The multi-layer brain network dataset was collected by Sun Yat-sen University [41]. The dataset includes three distinct groups, consisting of 51 deaf cases, 54 with tinnitus, and 42 healthy individuals. The study thoroughly analyzed their respective brain network function.

Resting-state EEG data can provide valuable information on neural processes. Extracting multi-layered brain network datasets from this data helps us understand how the brain functions. Complex datasets require advanced analysis methods, including varied subjects, electrodes, and frequency bands. These data examine the brain network dynamics in individuals with deafness, tinnitus, and those without hearing problems using 70 electrodes. Data acquisition and preprocessing are explained.

The dataset features nine different frequency bands, namely Delta, Theta, Alpha1, Alpha2, Beta1, Beta2, Beta3, Gamma1, and Gamma2. Pearson’s correlation coefficients calculate the interconnections of the electrodes for each frequency band. Based on EEG data, the network highlights significant disparities in neural networks between the three subject types. The dataset provides vital insights into the characteristics of brain networks in deafness and tinnitus patients compared to normal controls.

### Exploratory data analysis and visualization

Exploratory data analysis (EDA) is crucial in data science as it helps understand data patterns and gain insights. Visualizations and plots play a significant role in making complex data more accessible. In this EDA, we have employed various visualization techniques to elucidate the multi-layer brain network datasets obtained from EEG data. Graphical representations and data visualizations are essential for transforming intricate numerical data into easily understandable graphics. During this EDA process, we have tailored these visualizations to meet the unique requirements and characteristics of the multi-layer brain network datasets.

Histograms display how connection strengths are distributed within brain networks, such as the ’alpha1’ and ’alpha2’ connections in the normal category. These histograms group connection strengths into bins to reveal whether the networks consist predominantly of weak or strong connections. This visualizes the connections in the brain, where varying cell sizes indicate strength and color intensity represents patterns like clusters. Adjacency matrices offer a comprehensive network topology view by revealing how nodes are interconnected.

The clustering coefficient, based on triangle motifs or transitivity, measures how tightly knit a network is [42]. It is calculated as shown in Eq. 1:

1 $$\begin{aligned} C_i = \frac{2T_i}{k_i(k_i-1)}, \end{aligned}$$

where $C_i$ is the clustering coefficient of node *i*, $T_i$ is the number of triangles node *i* is part of and $k_i$ is the degree of node *i*.

**Fig. 1:**
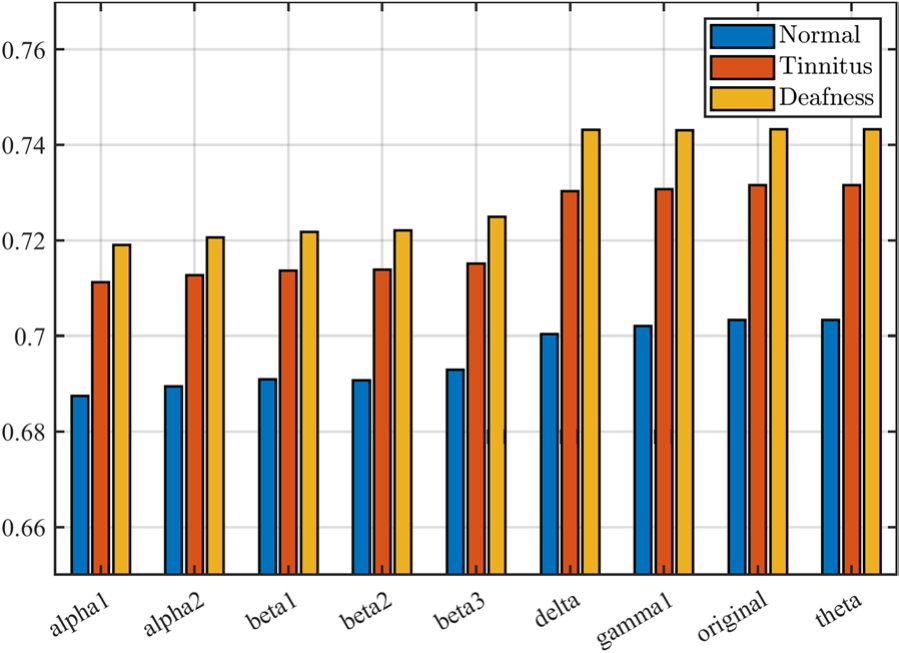
The clustering coefficients obtained from EEG scan data for three different auditory diagnosis cases: ”Normal”, ”Tinnitus”, and ”Deafness. Each bar in the plot represents the clustering coefficient for a specific scan channel and a particular diagnosis case

A node’s local clustering (per class) coefficient measures how well its neighbors are connected. It is calculated as shown in Eq. 2:

2 $$\begin{aligned} C_i = \frac{E_i}{k_i(k_i-1)/2}, \end{aligned}$$

where $C_i$ is the local clustering coefficient of node *i*, $E_i$ is the number of edges between the neighbors of node *i* and $k_i$ is the degree of node *i*. Figure 1 shows the clustering coefficients for the frequency band of the three cases and their corresponding averages. Provides a visual representation of clustering coefficients in EEG data that can be used as features to understand neural dynamics in auditory disorders better [43].

The clustering coefficient measures how tightly connected nodes are in a network, reflecting the degree of local connectivity in the EEG data. Across all scan channels, the ”Deafness” case tends to have slightly higher clustering coefficients compared to the ”Normal” and ”Tinnitus” cases. Additionally, the ”delta” scan channel exhibits the highest clustering coefficients among all cases. Furthermore, scan channels ”alpha1” and ”alpha2” demonstrate relatively high clustering coefficients for all diagnosis cases. The clustering coefficients obtained from the EEG scan data for the three cases of auditory diagnosis have ignited a strong motivation to explore machine learning for classification. These coefficients provide a unique perspective on local connectivity patterns within the EEG data, offering insights into the intricate relationships between different scan channels and diagnostic outcomes. The higher clustering coefficients associated with the ”Deafness” case across various scan channels hint at distinctive network properties that may indicate auditory disorders. This observation sparks curiosity about the underlying neural dynamics and its potential role in auditory conditions.

Moreover, the clustering coefficients shed light on the complex interplay between brain regions and their connectivity patterns. This intricate network of connections can be harnessed as valuable features for machine learning models to classify auditory diagnosis cases. Variations in clustering coefficients between different scan channels highlight the potential for discriminative features that capture the essence of each diagnostic category. We aim to create accurate machine learning models that classify individuals by auditory diagnosis for early detection to get accurate treatment.

## Ensemble learning classifiers

Identifying auditory impairments is challenging but crucial to improving quality of life. Efficient classification methods are needed to distinguish between affected and healthy individuals. Ensemble learning classifiers in audiology combine multiple models to improve classification accuracy and reduce variability and noise in auditory data [44, 45]. To effectively address auditory impairments, classification methods must be able to identify and understand subtle patterns and relationships within the data. Ensemble learning classifiers inherently capture diversity by incorporating distinct base classifiers, each specialized in recognizing specific patterns within the auditory features [46]. By using techniques such as bagging, boosting, and stacking, ensemble classifiers can better utilize the complex nature of auditory data [47]. Ensemble learning classifiers can accurately diagnose and treat auditory impairments using multiple models, providing better care for those with auditory disorders.

Given an annotated dataset $\{( \overrightarrow{X}_i, y_i)\}_{i=1}^{N}$, where $\overrightarrow{X}_i$ represents the input features and $y_i$ is the corresponding target label to build the ensemble.

### Extra Trees classifier

The Extra Trees classifier is known for effectively capturing complex patterns within intricate datasets [48]. Furthermore, brain networks provide a comprehensive display of functional connectivity across various neural regions. The Extra Trees classifier is a random forest algorithm that seeks to improve diversity and generalization by creating a group of decision trees [49]. It uses the input features and their corresponding target labels to build the ensemble, which involves training several decision trees. A single decision tree in the Extra Trees ensemble is grown using a random subset of features and training samples [50]. The splitting process aims to reduce the variance and overfitting inherent in individual trees [49]. The prediction of the *t*th tree is represented by $h_t(x)$, which is based on the input feature vector *x* provided. The ensemble prediction for an input *x* is calculated by averaging the predictions of all trees as presented in Eq. 3:

3 $$\begin{aligned} F(x) = \frac{1}{T} \sum _{t=1}^{T} h_t(x). \end{aligned}$$

The variable *T* represents the total number of trees in the ensemble. To create a prediction model, several decision trees are generated by training them in various random subsets of the data. The final prediction is made by combining the predictions of these trees. The randomness in the training process helps increase the model’s diversity and prevents overfitting [51]. This algorithm is simple and works as an ensemble. The algorithm’s simplicity and ensemble approach make it effective for various classification tasks, including diagnosing auditory disorders such as deafness, tinnitus, and normal auditory function. By enhancing the diversity among decision trees and considering the multi-view aspect of brain networks, the Extra Trees classifier holds the potential to enhance the accuracy and reliability of complex medical classifications. The combination of Extra Trees can lead to promising results in diagnosis.

### CatBoosting classifier

The CatBoosting classifier is a tool that can identify complex patterns within various datasets [52]. One area where it has been particularly useful is in analyzing the multi-view nature of brain networks. This allows for a detailed understanding of how different parts of the brain are connected and function together. The CatBoosting classifier is designed to improve an objective function by creating a sequence of decision trees. It achieves this by repeatedly building an ensemble of decision trees. The main function for CatBoosting can be expressed as in Eq. 4:

4 $$\begin{aligned} \text {Obj}(\theta ) = \sum _{i=1}^{N} L(y_i, F(x_i)) + \sum _{t=1}^{T} \Omega (f_t), \end{aligned}$$

where $\theta$ represents the model parameters, $F(x_i)$ is the ensemble prediction for input $x_i$, *L* is the loss function measuring the discrepancy between predicted and true labels, and $\Omega (f_t)$ is a regularization term for the *t*-th decision tree $f_t$. Each decision tree is constructed by recursively partitioning the feature space based on binary splits. The splitting process minimizes the loss function by determining optimal threshold values for each feature. Furthermore, CatBoosting uses a category-specific enhancement mechanism to effectively handle categorical characteristics [53]. When building trees with CatBoost, it is crucial to follow a step-by-step approach. Each new tree should be trained to learn the residuals of the previous ensemble’s predictions. This is done through gradient boosting, where new trees are added to reduce the gradient of the loss function. Combining the CatBoosting classifier with the multi-view approach can create a more accurate diagnostic model for identifying auditory disorders.

### Gradient Boosting classifier

The Gradient Boosting algorithm is a powerful classifier that can detect complex patterns in complicated datasets [54]. Combined with the multi-view analysis of brain networks, it comprehensively represents functional connectivity across different brain regions. Gradient Boosting combines weak learners to create a powerful predictive model [55]. The objective is to find an additive model as denoted in the Eq. 5:

5 $$\begin{aligned} F(x) = \sum _{t=1}^{T} \beta _t h_t(x), \end{aligned}$$

where *F*(*x*) is the final prediction for input *x*, *T* is the total number of weak learners, $\beta _t$ is the weight assigned to weak learner $h_t(x)$ which represents the output of the *t*th weak learner. Gradient Boosting minimizes the difference between predicted and actual labels using a loss function as denoted *L*(*y*, *F*(*x*)). This is done by adding weak learners to the ensemble in an iterative manner. In each iteration, a new weak learner denoted $h_t(x)$, is trained to estimate the negative gradient of the loss function related to the predictions of the current ensemble. This negative gradient is represented by the Eq. 6:

6 $$\begin{aligned} -\frac{\partial L(y, F(x))}{\partial F(x)}. \end{aligned}$$

The weight $\beta _t$ is determined by minimizing the loss function when the new weak learner is presented as in Eq. 7:

7 $$\begin{aligned} \beta _t = \arg \min _{\beta } \sum _{i=1}^{N} L(y_i, F_{t-1}(x_i) + \beta h_t(x_i)), \end{aligned}$$

where $F_{t-1}(x_i)$ represents the ensemble’s prediction up to the $(t-1)$-th iteration. The ensemble prediction is then updated as in Eq. 8:

8 $$\begin{aligned} F_t(x) = F_{t-1}(x) + \beta _t h_t(x). \end{aligned}$$

Gradient Boosting uses weak learners and adjusts their weights to create an accurate predictive model. This method combines the strengths of multiple learners for more accurate results [56]. To diagnose auditory disorders effectively, use gradient boosting and multi-view network data in the proposed model.

### Random Forest classifier

The Random Forest algorithm is a trustworthy machine learning technique that uses ensemble learning to achieve accurate classifications [57]. This classifier is essential for managing large and complex data, which has been widely used in various fields [50, 58, 59]. The Random Forest classifier is an ensemble learning technique that aims to improve the predictive accuracy and robustness of individual decision trees [60]. The proposed algorithm constructs an ensemble of decision trees to make predictions. In a Random Forest ensemble, each decision tree is built by dividing the feature space using binary splits. To introduce randomness, only a subset of features is considered for each split during training. The output of a decision tree for a given input feature vector is denoted as $h_t(x)$. The ensemble prediction for input *x* is obtained by aggregating the predictions of all trees as presented in Eq. 3. The Random Forest algorithm mitigates the overfitting often associated with individual decision trees. The ensemble approach leverages the diverse perspectives of individual trees to improve generalization performance. By introducing randomness in the feature selection process and aggregating the predictions, Random Forest balances bias and variance, leading to robust and accurate predictions.

## Experimental modeling hypothesis

Developing a robust classification model to differentiate between individuals with auditory disorders requires the integration of Multi-View Brain Networks. These complex neural connectivity patterns captured by Multi-View Brain Networks provide a rich source of information for the classification process. Therefore, leveraging these multi-dimensional brain network representations can potentially build an effective and accurate classification model. This model can distinguish between different auditory diagnosis classes and underlying neural mechanisms associated with these conditions. This can be a significant step in enabling early diagnosis and treatment of auditory disorders.

### Evaluation metrics

To rigorously assess the performance and efficacy of our classification model, we employ a comprehensive set of evaluation metrics, each offering unique insights into its capabilities. These metrics include:

1. *Accuracy:* The main measure for performance by computing the proportion of instances that are correctly classified and provides an overall model performance.
2. *Cross-validation accuracy (CVA):* An important metric that measures the performance of a model across multiple iterations of cross-validation. It ensures that the classification model is consistent and can make accurate predictions across diverse data.
3. *Precision:* A measure that quantifies the proportion of true positive predictions relative to the total positive predictions when evaluating the model’s ability to make accurate positive classifications.
4. *Recall:* The appropriate measure to evaluates the model’s ability to identify all actual positive instances by calculating the proportion of true positives identified correctly.
5. *F1-score:* A measure balances precision and recall by considering false positives and negatives, providing a robust measure of accuracy.
6. *Kappa:* A measure provides a reliable measure of classification understanding between the model’s predictions and actual labels.
7. *MCC:* A useful metric for imbalanced data. It measures the correlation between a model’s predictions and the actual labels.
8. *Zero-one loss:* A measures for the ratio of misclassified instances and highlights the severity of misclassifications.
9. *Hamming loss:*A measures computes the proportion of incorrectly assigned labels, providing insight into multi-class classification accuracy.

We use Multi-View Brain Networks to classify auditory diagnostic cases through a hypothesis-based methodology. By incorporating essential metrics and diverse evaluation techniques, we aim to conduct a thorough assessment of the classification model’s effectiveness by leveraging a comprehensive suite of evaluation metrics (Table 1).

**Table 1:** The hypothetical formulation of the model’s evaluation metrics based on the classifier outputs and sample true label

Measure	Mathematical formula
Accuracy	$\text {Accuracy} = \frac{TP + TN}{\text {Total samples}}$
Precision	$\text {Precision} = \frac{TP}{TP + FP}$
Recall (sensitivity)	$\text {Recall} = \frac{TP}{TP + FN}$
F1-score	$\text {F1-score} = \frac{2 \times Precision \times recall}{Precision + Recall}$
Kappa	$\text {Kappa} = \frac{\text {Accuracy} - \text {Expected accuracy}}{1 - \text {Expected accuracy}}$
MCC	$\text {MCC} = \frac{TP \times TN - FP \times FN}{\sqrt{(TP + FP) \times (TP + FN) \times (TN + FP) \times (TN + FN)}}$
Zero-one loss	$\text {Zero-one L} = \frac{\text {Number of Misclassified samples}}{\text {Total samples}}$
Hamming loss	$\text {HL} = \frac{\text {Number of incorrect label assignments}}{\text {Total number of labels}}$

### Proposed modeling: experimental setup

To create accurate classification models for individuals with auditory disorders, a comprehensive experimental setup was designed to ensure reliable and meaningful results. This involved fine-tuning the hyperparameters and optimizing the preprocessing steps to enhance the performance of the ensemble learning model. The study used extra trees, random forest, gradient boosting, and CatBoost due to their proven efficacy in complex classification tasks. Due to their intrinsic capacity to capture complex relationships within the data, these models were considered suitable for diagnosing auditory disorders from Multi-View Brain Networks data. An essential step in the experimental process was to preprocess the raw data, improving its suitability for model training and evaluation. PCA was used to reduce the dimensionality of brain connectivity data effectively. This technique reduces feature space while retaining influential patterns. The data were then divided into training and testing subsets for model evaluation. Normalization was applied as an essential preprocessing step to ensure all features were on the same scale. This minimized the influence of varying scales on model performance and accelerated the convergence of iterative optimization algorithms. As a result, the training process was more efficient and effective. Table 2 shows the selected hyperparameters for each model after tuning.

### Wilcoxon signed-rank test

The Wilcoxon signed-rank test is a non-parametric statistical test used to determine whether there are statistically significant differences between two related or paired groups or conditions [61]. It is widely used for experiment evaluation purposes such as in [62–64]. This test is particularly useful when the assumptions of normality and equal variances still need to be met or when dealing with ordinal or non-normally distributed data. The Wilcoxon signed-rank test compares two sets of related or paired observations [65]. These paired observations can represent measurements taken before and after an intervention or any other related data points.

**Table 2:** Hyperparameter settings for each model

Model	Hyperparameters
Extra Trees	N Classifiers=100 max depth=none
Random Forest	N Classifiers=100 max depth=none
Gradient Boosting	Learning rate=0.001 max depth=10 N Classifiers=50
CatBoost	Learning rate=0.021 max depth=8 N Classifiers=100

Hyperparameters were fine-tuned through grid search and cross-validation technique (*K*=5 folds) to optimize performance and improve generalization while mitigating overfitting. The combined approach of preprocessing, hyperparameter tuning, and ensemble learning was tested. Models trained on preprocessed data with optimized hyperparameters showed better performance in classifying individuals with auditory disorders.

## Experimental findings and results

In audiology, it can be challenging to differentiate individuals who suffer from hearing loss or tinnitus from normal cases. It demands careful identification and comprehensive analysis, which cannot be underestimated. Patients suffering from auditory disorders experience a significant reduction in their quality of life. Therefore, it is essential to develop accurate and efficient classification methods. This study presents a novel approach that utilizes Multi-view Brain Networks data and various ensemble learning technologies. Analyzing brain connectivity data obtained from EEG measurements can be difficult due to the many dimensions involved. This study effectively overcomes the challenge by implementing PCA. PCA can decrease the number of dimensions of features but also enhance the accuracy of the diagnosis process [66].

### Classification results

Table 3 provides a comprehensive overview of the performance of each of the four ensemble learning models per class. As an instance, the Extra Trees model indicates CVA rates of 89.58% for individuals with deafness and 86.96% for those tinnitus cases. These metrics reflect the model’s capability to diagnose the respective classes accurately. Moreover, the recall values, which measure the model’s accuracy in correctly identifying instances from each class, are closely aligned with the precision values.

**Table 3:** Classification results for each model per class

Model	Class	Precision (%)	Recall (%)	F1-score
Extra Trees	Normal	91.30	92.65	91.97
	Deafness	89.58	87.16	88.36
	Tinnitus	86.96	88.24	87.59
CatBoost	Normal	92.54	91.18	91.85
	Deafness	88.59	89.19	88.89
	Tinnitus	86.86	87.50	87.18
Random Forest	Normal	89.51	94.12	91.76
	Deafness	92.20	87.84	89.97
	Tinnitus	87.50	87.50	87.50
Gradient Boosting	Normal	92.48	90.44	91.45
	Deafness	87.92	88.51	88.22
	Tinnitus	86.96	88.24	87.59

When evaluating the performance of different models, the F1-score is a useful metric that considers both precision and recall. Specifically, it measures how well a model balances correct classifications by minimizing false positives and negatives. For instance, the Gradient Boosting model has an F1-score of 88.22 for the deafness class, indicating its ability to achieve this balance effectively.

**Table 4:** Comprehensive classification results for proposed models

Model	Extra Trees	CatBoost	Random Forest	Gradient Boosting
CVA (%)	89.52	89.52	89.76	89.05
Precision (%)	89.56	89.33	89.74	89.11
Recall (%)	89.52	89.29	89.76	89.05
F1-score	89.49	89.31	89.74	89.08
Kappa	0.84244	0.79155	0.83525	0.81382
MCC	0.84322	0.79363	0.83533	0.81385
Zero-one loss	0.10476	0.13810	0.10952	0.12381
Hamming loss	0.10476	0.13810	0.10952	0.12381
Mean	89.52	89.37	89.75	89.02
Variance	0.00015	0.00067	0.00031	0.00029

Table 4 presents the evaluation results of the proposed models for classifying individuals with auditory disorders. The Extra Trees model demonstrated a balanced performance with accuracy, precision, recall, and F1-score of approximately 89.5%, indicating consistent accuracy across different classes. Moreover, CatBoost had slightly lower precision and recall values within the range of 89%. However, the Random Forest model outperformed the other models with accuracy and F1-score of 89.76%, accompanied by corresponding precision and recall values of 89.74%. This shows a solid ability to minimize false positives and false negatives. Although Gradient Boosting had slightly lower metrics, it maintained competitive precision, recall, and F1-score scores above 89%. Also, Table 4 displays the performance metrics that evaluate the effectiveness of the proposed models in handling the classification of auditory disorders. This analysis gives a complete overview of their performance. Interestingly, Extra Trees had the highest Kappa and MCC values, reaching over 0.84. This highlights its strong ability to capture the underlying patterns accurately. Extra trees consistently displayed low values for zero-one loss and hamming loss, with a score of 0.10476, highlighting its ability to minimize misclassifications across multiple classes. The Random Forest algorithm demonstrated exceptional performance, achieving Kappa and MCC values above 0.83. This highlights its ability to classify data and minimize prediction errors accurately. Even though CatBoost and Gradient Boosting had slightly lower metrics than Extra Trees and Random Forest, they still had impressive Kappa and MCC values, exceeding 0.79. Furthermore, their zero-one loss and hamming loss results were slightly elevated but fell within an acceptable range. Based on the results, it is clear that ensemble learning models were successful in multiple performance metrics. In particular, Extra Trees and Random Forest demonstrated exceptional performance across all metrics, proving their ability to classify individuals with auditory disorders while accurately minimizing classification errors. We have included mean and variance values, developing our model performance evaluation. Mean values provide an important direction measure, showing the average performance across different cross-validation folds. For instance, the mean classification results range from 89.02 to 89.75%, demonstrating our models’ consistency. Variance values provide insights into the spread or variability of the results, highlighting the stability of our proposed methodology. Low variance values, such as 0.00015, suggest a narrow distribution of performance metrics, increasing the robustness and reliability of our classification models. This accurate examination of mean and variance values enhances the clarity and completeness of our evaluation, contributing to a more slight understanding of our models’ performance characteristics.

### Proposed study findings and discussion

**Fig. 2:**
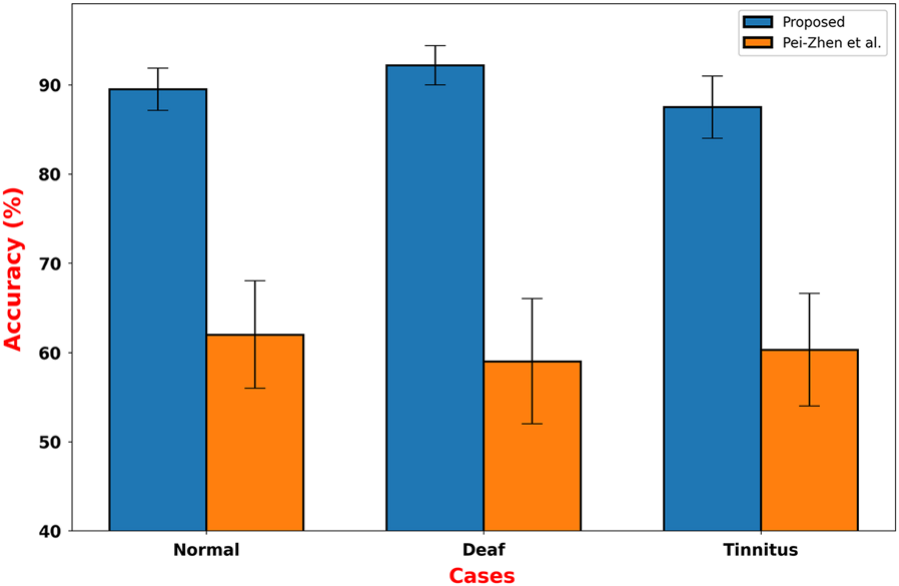
Over 20 runs, the measures mean accuracy represented by bars with standard deviation as error bars for the proposed model and Pei-Zhen et al. using random forest classifier classification results

To accurately diagnose auditory disorders, evaluating the effectiveness of different methodologies is essential. Recent advancements in this field have the potential to significantly improve diagnostic and therapeutic approaches, ultimately leading to better patient care. Pei-Zhen et al. [25] conducted a study using a Random Forest algorithm-based classification model to enhance the precision of auditory disorder diagnoses. We performed a comparative analysis of our proposed methodology and the findings presented by Pei-Zhen et al. in their study. Our results are shown in Fig. 2, where the bars and error indicators illustrate the superiority of our approach in terms of both performance and stability. Furthermore, our proposed model outperformed Pei-Zhen et al.’s study, indicating significant improvement in classification accuracy. This promising result could lead to the development of refined diagnostic frameworks and more effective therapeutic interventions for auditory disorders.

Table 5 compares the proposed approach and Pei-Zhen et al.’s method, indicating that the difference is significant with a *p*-value of 0.0001 and a test statistic of 210. There is a significant difference in performance between the two methods, with a test statistic of 210 and a *p*-value of 0.0001. Our approach outperforms Pei-Zhen et al.’s method significantly. The test statistic reflects the magnitude of this difference, and the low p-value indicates a high level of significance, further supporting the superiority of our approach.

**Table 5:** Comparison of proposed approach vs. Pei-Zhen et al.’s method

Method pair	Test statistic	*p*-value	Result
Proposed vs. Pei-Zhen et al.	${{210}}$	${{0.0001}}$	Significant

Table 6 provides the statistical significance of proposed different classifiers used in diagnosing auditory disorders with Multi-View Brain Network data.

**Table 6:** Wilcoxon signed-rank test results for classifier comparisons

Classifier pair	Test statistic	*p*-value	Result
**Random Forest vs. Extra Trees**	**22.5000**	**0.0296**	**Significant**
**Random Forest vs. Gradient Boosting**	**0.001**	**0.0039**	**Significant**
**Random Forest vs. CatBoost**	**12.200**	**0.0002**	**Significant**
Extra Trees vs. Gradient Boosting	48.0000	1.0000	Not significant
**Extra Trees vs. CatBoost**	**4.0000**	**0.0156**	**Significant**
**Gradient Boosting vs. CatBoost**	**8.0000**	**0.0010**	**Significant**

Using Wilcoxon signed-rank test to indicate if exists a statistically significant and reliability for differences in scored accuracy of each model, according to the test outcomes only significant comparisons with *p*-value<0.5 are marked Bold

It presents the outcomes of the Wilcoxon signed-rank test, which includes test statistics, *p*-values, and corresponding significance classifications. This information sheds light on the effectiveness of each classifier and their differences in the diagnosis of auditory disorders. The comparison between the ”Random Forest” classifier and other classifiers such as ”Extra Trees”, ”Gradient Boosting”, and ”CatBoost” showed statistically significant differences in accuracy. The test statistic was 22.5000 with a *p*-value of 0.0296 for ”Extra Trees”, 0.001 with a *p*-value of 0.0039 for ”Gradient Boosting”, and 12.200 with a *p*-value of 0.0002 for ”CatBoost”. These results highlight the significant differences in diagnostic performance between ”Random Forest” and each of these classifiers, emphasizing the critical nature of classifier selection in influencing diagnostic accuracy. On the other hand, the comparison between ”Extra Trees” and ”Gradient Boosting” resulted in a ”Not significant” outcome with a test statistic of 48.0000 and a *p*-value of 1.0000. This implies a lack of statistically discernible differences in accuracy between these two classifiers, suggesting a certain level of similarity in their diagnostic performance. The comparisons between ”Extra Trees” and ”CatBoost” (test statistic = 4.0000, *p*-value = 0.0156) and ”Gradient Boosting” and ”CatBoost” (test statistic = 8.0000, *p*-value = 0.0010) produced ”Significant” results, indicating significant disparities in diagnostic accuracy between these classifier pairs. These findings highlight the important impact of classifier selection on diagnostic outcomes, as the choice between ”Extra Trees” and ”CatBoost” or ”Gradient Boosting” and ”CatBoost” significantly influences the overall diagnostic performance in auditory disorders. In closing, the outcomes of the Wilcoxon signed-rank test provide a slight understanding of the statistical significance underpinning the comparative performance of diverse classifiers in the diagnosis of auditory disorders using Multi-View Brain Network data. The discerned ”Significant” disparities in accuracy, presented in the comparisons involving Random Forest with Extra Trees, Gradient Boosting, and CatBoost, highlight the significant differences in performing Random Forest compared to these classifiers. Conversely, the observed ”Not Significant” result for the Extra Trees vs. Gradient Boosting pairing explains the absence of a discernible difference in accuracy, emphasizing the similarity in their performance. The additional ”Significant” findings for the Extra Trees vs. CatBoost and Gradient Boosting vs. CatBoost comparisons highlight the essential role of classifier selection in auditory disorder diagnosis. These results highlight the significance of robust statistical analyses in guiding the selection of the most effective models, presenting a promising avenue for advancing early detection and personalized treatment strategies and contributing to reducing the quality of life for individuals with auditory disorders.

## Conclusion and future scope

The main aim of this study is to address the essential challenges associated with differentiating individuals with hearing impairments, such as deafness and tinnitus, from those with normal hearing. The study has contributed significantly to the field by presenting an improved approach for accurately classifying auditory disorders. We achieved this by using Multi-View Brain Network data and leveraging advanced machine learning algorithms, including Random Forest, Extra Trees, Gradient Boosting, and CatBoost. Our research findings have revealed that our proposed model performs exceptionally well across multiple evaluation metrics, such as accuracy, precision, recall, and F1-score. Notably, the Random Forest model has shown outstanding accuracy, precision, and F1-score values, highlighting its effectiveness in differentiating between different subject groups. These promising results have the potential to revolutionize the early detection and personalized treatment of auditory disorders, leading to better patient outcomes and an enhanced quality of life. Our study investigates the performance of different classifiers in diagnosing auditory disorders and compares them using the Wilcoxon signed-rank test. This statistical analysis emphasizes the importance of selecting the appropriate classifier for accurate diagnosis. The significant differences in accuracy between various classifiers highlight the critical need to choose the right model for maximizing diagnostic accuracy. In the future, more research can be conducted to improve this work by including other data sources, such as neuroimaging data or genetic markers, which can increase the model’s predictive power. Testing the model’s reliability among different populations is also recommended to ensure its effectiveness in various clinical settings. As deep learning and neuroscience advance, there is a great opportunity to refine and enhance the model’s methods, which can lead to significant progress in audiology and auditory disorder diagnosis.

## Data Availability

The authors declare the source code and any additional materials are accessible upon request.

## References

[1] Henry JA, Reavis KM, Griest SE, Thielman EJ, Theodoroff SM, Grush LD, Carlson KF (2020). Tinnitus: an epidemiologic perspective. Otolaryngol Clin N Am.

[2] Chadha S, Kamenov K, Cieza A (2021). The world report on hearing, 2021. Bull World Health Organ.

[3] Jin Y, Gao Y, Guo X, Wen J, Li Z, Jin Z (2022) Earhealth: an earphone-based acoustic otoscope for detection of multiple ear diseases in daily life. In: Proceedings of the 20th annual international conference on mobile systems, applications and services, pp. 397–408

[4] Tang D, Li H, Chen L (2019) Advances in understanding, diagnosis, and treatment of tinnitus. Hearing loss: mechanisms, prevention and cure. Springer, Singapore, p 109–128 10.1007/978-981-13-6123-4_7 30915704

[5] Gesuita L, Karayannis T (2023) The beautiful brain: communicating fundamental neuroscience through masterpieces of art. Wiley Online Library 10.1002/1873-3468.14604 36929370

[6] Azizi SA (2022). Monoamines: dopamine, norepinephrine, and serotonin, beyond modulation,“switches” that alter the state of target networks. Neuroscientist.

[7] Lindsay G (2021). Models of the mind: how physics, engineering and mathematics have shaped our understanding of the brain.

[8] Bassett DS, Bullmore ET (2009). Human brain networks in health and disease. Curr Opin Neurol.

[9] Sanei S, Chambers JA (2021). EEG signal processing and machine learning.

[10] Esfahlani FZ, Jo Y, Puxeddu MG, Merritt H, Tanner JC, Greenwell S, Patel R, Faskowitz J, Betzel RF (2021). Modularity maximization as a flexible and generic framework for brain network exploratory analysis. Neuroimage.

[11] Zhu Y, Li X, Qiao Y, Shang R, Shi G, Shang Y, Guo H (2021). Widespread plasticity of cognition-related brain networks in single-sided deafness revealed by randomized window-based dynamic functional connectivity. Med Image Anal.

[12] Alencar CD, Butler BE, Lomber SG (2019). What and how the deaf brain sees. J Cogn Neurosci.

[13] Dell Ducas K, Senra Filho ACdS, Silva PHR, Secchinato KF, Leoni RF, Santos AC (2021). Functional and structural brain connectivity in congenital deafness. Brain Struct Funct.

[14] Guidetti G, Guidetti R, Quaglieri S (2021). Sport as a factor in improving visual spatial cognitive deficits in patients with hearing loss and chronic vestibular deficit. Audiol Res.

[15] Rodger H, Lao J, Stoll C, Richoz A-R, Pascalis O, Dye M, Caldara R (2021) The recognition of facial expressions of emotion in deaf and hearing individuals. Heliyon 7(5):07018 10.1016/j.heliyon.2021.e07018 PMC8141778 34041389

[16] Timmer BH, Bennett RJ, Montano J, Hickson L, Weinstein B, Wild J, Ferguson M, Holman JA, LeBeau V, Dyre L (2023) Social-emotional well-being and adult hearing loss: clinical recommendations. Int J Audiol 1–12 10.1080/14992027.2023.2190864 36960799

[17] Singh A, Smith PF, Zheng Y (2023). Targeting the limbic system: insights into its involvement in tinnitus. Int J Mol Sci.

[18] Khan RA, Sutton BP, Tai Y, Schmidt SA, Shahsavarani S, Husain FT (2021). A large-scale diffusion imaging study of tinnitus and hearing loss. Sci Rep.

[19] Weisz N, Moratti S, Meinzer M, Dohrmann K, Elbert T (2005). Tinnitus perception and distress is related to abnormal spontaneous brain activity as measured by magnetoencephalography. PLoS Med.

[20] Wimalarathna H, Ankmnal-Veeranna S, Allan C, Agrawal SK, Allen P, Samarabandu J, Ladak HM (2021). Comparison of machine learning models to classify auditory brainstem responses recorded from children with auditory processing disorder. Comput Methods Programs Biomed.

[21] Chen Y-C, Li X, Liu H, Long X, Liu B, Zhou F, Chen Y-F (2020). Auditory network alterations in tinnitus revealed by resting-state functional connectivity. Neuroscience.

[22] Smith AK, Jones KD (2018). Neuroimaging and plasticity in deafness. Neuroscientist.

[23] Li X, Morgan PS, Ashburner J (2019). Machine learning on brain imaging data: a comprehensive tutorial. Neuroimage.

[24] Johnson HJ, Paul D, Abed-Meraim K (2017). Multi-modal data fusion in neuroimaging: Overview and challenges. Brain Informatics.

[25] Li P-Z, Huang L, Wang C-D, Li C, Lai J-H (2019). Brain network analysis for auditory disease: a twofold study. Neurocomputing.

[26] Xu L, Wang C-D, Liang M-J, Cai Y-X, Zheng Y-Q (2018) Brain network regional synchrony analysis in deafness. BioMed Res Int 2018 1–11 10.1155/2018/6547848 PMC5949203 29854776

[27] Kurmukov A, Dodonova Y, Zhukov LE (2017) Machine learning application to human brain network studies: a kernel approach. In: Models, algorithms, and technologies for network analysis: NET 2016, Nizhny Novgorod, Russia, May 2016 6, pp 229–249. Springer

[28] Shoushtarian M, Alizadehsani R, Khosravi A, Acevedo N, McKay CM, Nahavandi S, Fallon JB (2020). Objective measurement of tinnitus using functional near-infrared spectroscopy and machine learning. PLoS ONE.

[29] Hu J, Cui J, Xu J-J, Yin X, Wu Y, Qi J (2021) The neural mechanisms of tinnitus: a perspective from functional magnetic resonance imaging. Front Neurosci 15:621145 10.3389/fnins.2021.621145 PMC7905063 33642982

[30] Thomas Yeo B, Krienen FM, Sepulcre J, Sabuncu MR, Lashkari D, Hollinshead M, Roffman JL, Smoller JW, Zöllei L, Polimeni JR (2011). The organization of the human cerebral cortex estimated by intrinsic functional connectivity. J Neurophysiol.

[31] Yeo BT, Krienen FM, Sepulcre J, Sabuncu MR, Lashkari D, Hollinshead M, Roffman JL, Smoller JW, Zöllei L, Polimeni JR, et al (2011) The organization of the human cerebral cortex estimated by intrinsic functional connectivity. J Neurophysiol 10.1152/jn.00338.2011 PMC3174820 21653723

[32] Zhang J, Abiose O, Katsumi Y, Touroutoglou A, Dickerson BC, Barrett LF (2019). Intrinsic functional connectivity is organized as three interdependent gradients. Sci Rep.

[33] Bergmann E, Zur G, Bershadsky G, Kahn I (2016) The organization of mouse and human cortico-hippocampal networks estimated by intrinsic functional connectivity. Cereb Cortex 1–16 10.1093/cercor/bhw327 PMC5193145 27797832

[34] Kumar A, Tewari N, Kumar R (2021) Study towards the analytic approach for human computer interaction using machine learning. Int J Anal Exp Modal Anal 11

[35] Gowtham KM, Ganesh C, Nalawade SS, Davenport EM, Wagner B, Kim WH, Maldjian JA (2020). Brainnet: inference of brain network topology using machine learning. Brain Connect.

[36] Liu M, Zhang Z, Dunson DB (2021). Graph auto-encoding brain networks with applications to analyzing large-scale brain imaging datasets. Neuroimage.

[37] Ilinka I, Trivodaliev K, Kalajdziski S, Zanin M (2021). Statistical and machine learning link selection methods for brain functional networks: Review and comparison. Brain Sci.

[38] Bi X, Zhao X, Huang H, Chen D, Ma Y (2020). Functional brain network classification for Alzheimer’s disease detection with deep features and extreme learning machine. Cogn Comput.

[39] Zuo Q, Lei B, Shen Y, Liu Y, Feng Z, Wang S (2021) Multimodal representations learning and adversarial hypergraph fusion for early Alzheimer’s disease prediction. In: Pattern recognition and computer vision: 4th Chinese Conference, PRCV 2021, Beijing, China, October 29–November 1, 2021, Proceedings, Part III 4, pp. 479–490. Springer

[40] Wang S, Chen Z, You S, Wang B, Shen Y, Lei B (2022). Brain stroke lesion segmentation using consistent perception generative adversarial network. Neural Comput Appl.

[41] Multi-view Brain Networks (2020) UCI Machine Learning Repository. https://doi.org/10.24432/C5JS62

[42] Arrigo F, Higham DJ, Tudisco F (2020). A framework for second-order eigenvector centralities and clustering coefficients. Proc R Soc A.

[43] Kılıç B, Aydın S (2022). Classification of contrasting discrete emotional states indicated by EEG based graph theoretical network measures. Neuroinformatics.

[44] Manta O, Sarafidis M, Schlee W, Mazurek B, Matsopoulos GK, Koutsouris DD (2023). Development of machine-learning models for tinnitus-related distress classification using wavelet-transformed auditory evoked potential signals and clinical data. J Clin Med.

[45] Lenatti M, Moreno-Sánchez PA, Polo EM, Mollura M, Barbieri R, Paglialonga A (2022). Evaluation of machine learning algorithms and explainability techniques to detect hearing loss from a speech-in-noise screening test. Am J Audiol.

[46] Tanveer M, Rastogi A, Paliwal V, Ganaie M, Malik A, Del Ser J, Lin C-T (2023) Ensemble deep learning in speech signal tasks: a review. Neurocomputing 126436

[47] Mohammed A, Kora R (2023) A comprehensive review on ensemble deep learning: opportunities and challenges. J King Saud Univ-Comput Inf Sci 35(2):757–774

[48] Gupta S, Arango-Argoty G, Zhang L, Pruden A, Vikesland P (2019). Identification of discriminatory antibiotic resistance genes among environmental resistomes using extremely randomized tree algorithm. Microbiome.

[49] Sagi O, Rokach L (2018). Ensemble learning: a survey. Wiley Interdiscipl Rev Data Mining Knowl Discov.

[50] Ghiasi MM, Zendehboudi S (2021). Application of decision tree-based ensemble learning in the classification of breast cancer. Comput Biol Med.

[51] Jiang M, Liu J, Zhang L, Liu C (2020). An improved stacking framework for stock index prediction by leveraging tree-based ensemble models and deep learning algorithms. Physica A.

[52] Hussain S, Mustafa MW, Jumani TA, Baloch SK, Alotaibi H, Khan I, Khan A (2021). A novel feature engineered-catboost-based supervised machine learning framework for electricity theft detection. Energy Rep.

[53] Rahim A, Zhong Y, Ahmad T, Ahmad S, Pławiak P, Hammad M (2023). Enhancing smart home security: anomaly detection and face recognition in smart home iot devices using logit-boosted cnn models. Sensors.

[54] Rawat R, Mahor V, Chirgaiya S, Shaw RN, Ghosh A (2021) Analysis of darknet traffic for criminal activities detection using tf-idf and light gradient boosted machine learning algorithm. In: Innovations in Electrical and Electronic Engineering: Proceedings of ICEEE 2021, pp. 671–681. Springer

[55] Wang J, Li P, Ran R, Che Y, Zhou Y (2018). A short-term photovoltaic power prediction model based on the gradient boost decision tree. Appl Sci.

[56] Feng D-C, Liu Z-T, Wang X-D, Chen Y, Chang J-Q, Wei D-F, Jiang Z-M (2020). Machine learning-based compressive strength prediction for concrete: an adaptive boosting approach. Constr Build Mater.

[57] AlJame M, Ahmad I, Imtiaz A, Mohammed A (2020). Ensemble learning model for diagnosing COVID-19 from routine blood tests. Inform Med Unlocked.

[58] Kiangala SK, Wang Z (2021). An effective adaptive customization framework for small manufacturing plants using extreme gradient boosting-xgboost and random forest ensemble learning algorithms in an industry 4.0 environment. Mach Learn Appl.

[59] Mishra AK, Paliwal S (2023). Mitigating cyber threats through integration of feature selection and stacking ensemble learning: the lgbm and random forest intrusion detection perspective. Clust Comput.

[60] Dumitrescu E, Hué S, Hurlin C, Tokpavi S (2022). Machine learning for credit scoring: improving logistic regression with non-linear decision-tree effects. Eur J Oper Res.

[61] Mishra P, Pandey CM, Singh U, Keshri A, Sabaretnam M (2019). Selection of appropriate statistical methods for data analysis. Ann Card Anaesth.

[62] Ahmed MAO, Didaci L, Lavi B, Fumera G (2017) Using diversity for classifier ensemble pruning: an empirical investigation. Theoret Appl Inform 29(1–2):25–39

[63] Ahmed MA, Didaci L, Fumera G, Roli F (2015) An empirical investigation on the use of diversity for creation of classifier ensembles. In: Multiple Classifier Systems: 12th International Workshop, MCS 2015, Günzburg, Germany, June 29–July 1, 2015, Proceedings 12, pp. 206–219. Springer

[64] Khalifa HS, Wahhab H, Alanssari A, Khfagy MOA (2019). Fingerprint segmentation approach for human identification. Appl Math.

[65] Taheri S, Hesamian G (2013). A generalization of the Wilcoxon signed-rank test and its applications. Stat Pap.

[66] Attallah O (2020). An effective mental stress state detection and evaluation system using minimum number of frontal brain electrodes. Diagnostics.

